# The Impact of a Smartphone Reminder Application on Artificial Tear Adherence in Dry Eye Disease

**DOI:** 10.3390/jcm15166369

**Published:** 2026-08-18

**Authors:** Moonisah Ayaz, Gracelynn De Barros, Khadija Ahmed, Sònia Travé Huarte, Alec Kingsnorth, James S. Wolffsohn

**Affiliations:** 1 School of Optometry, Aston University, Birmingham B4 7ET, UKs.trave-huarte@aston.ac.uk (S.T.H.); 2 Wolffsohn Research Ltd., Lisburn BT28 2WG, UK

**Keywords:** dry eye disease, compliance, application, smartphone

## Abstract

**Background**: Dry eye disease (DED) impairs quality of life. Artificial tears are the first-line treatment, but compliance is generally poor. Patients forget to instil drops or underestimate the importance of regular use. Mobile health (mHealth) applications improve adherence in other chronic conditions, but their role in DED remains unclear. This study evaluated the effectiveness of a smartphone reminder application in improving compliance to a four-times-daily artificial tear regimen and the associated symptom relief among patients with DED. **Methods**: A masked randomised crossover trial was conducted in 29 women with DED (mean ± SD age 21.2 ± 3.0 years). Participants completed two 14-day study phases: one with app-based reminders and one without, separated by a 7-day washout period. The primary outcome was mean daily drop frequency. Secondary outcomes were self-reported symptom frequency and severity assessed using the Symptom Assessment iN Dry Eye (SANDE). **Results**: The mean daily drop frequency was higher during the app phase compared with the non-app phase (*F* = 22.906, *p* < 0.001) but declined in both phases over time (*F* = 3.023, *p* < 0.001). Symptom frequency did not differ between phases but decreased over time (*F* = 1.993, *p* = 0.021). Symptom severity remained unchanged with no significant effects by phase or time. Baseline clinical measures did not predict drop use frequency or app-related improvement (*p* > 0.05). **Conclusions**: The reminder application increased short-term compliance to artificial tear use, but there was no corresponding reduction in symptoms or signs over two weeks’ usage. Compliance waned over time despite active reminders, suggesting that prompts alone are insufficient for sustaining behaviour change. Future digital interventions for DED should incorporate strategies to enhance motivation, support long-term engagement, and provide personalised education.

## 1. Introduction

Dry eye disease (DED) is a highly prevalent ocular surface disorder that diminishes quality of life [1]. Global prevalence is estimated at 34.6%, affecting approximately one third of adults in the United Kingdom [2,3]. The condition occurs more frequently in women, older adults, and individuals of East Asian ancestry, contributing substantially to healthcare system burden [4,5].

The TFOS DEWS III report defines DED as a multifactorial disease of the tear film and ocular surface, characterised by loss of homeostasis and driven by tear film instability, inflammation, and ocular surface damage [6]. Clinically, DED can be subclassified into tear film deficiencies, eyelid anomalies, and ocular surface abnormalities, an aetiological framework that guides management [6,7].

Symptoms including visual disturbance, foreign body sensation, photophobia, and ocular fatigue can significantly impair reading, driving, screen use, and occupational performance [8]. Anxiety, depression, and sleep disturbance are more prevalent in DED populations, and the condition must therefore be understood not only by its clinical signs but also by its broader effects on daily function and wellbeing [9,10].

Artificial tears are lubricating eye drops used as first-line treatment for irritation and dryness associated with tear-deficient dry eye disease [11]. They supplement tear volume, stabilise the tear film, and reduce the concentration of inflammatory mediators on the ocular surface [12,13]. Evidence-based guidance recommends instillation four times daily to achieve clinically meaningful symptom relief, with sustained improvement typically observed after one month of regular use [14]. Real-world adherence, however, is poor. Patients forget doses, lack environmental cues, and question the need for treatment when symptoms are absent [11,15,16].

Forgetfulness is the most cited barrier and is modifiable [17]. Many patients use drops only when symptoms worsen or stop when immediate relief is not perceived, failing to maintain tear film homeostasis [18,19]. Regular preventive use improves objective signs, yet patients often treat artificial tears as ‘rescue therapy’ rather than maintenance [20]. Health literacy is also an important factor; understanding that artificial tears act to stabilise the tear film rather than simply provide symptomatic relief may promote regular use [21]. In eye care, limited health literacy has been linked to poorer adherence and adverse clinical outcomes [22].

Such barriers are effectively conceptualised using two behavioural frameworks. The COM-B model proposes that behaviour requires capability, opportunity, and motivation [23]. In DED, compliance demands the psychological capability to remember dosing, the opportunity provided by cues and routines, and the motivation to continue when symptoms are mild or absent. The Health Belief Model (HBM) adds that behaviour is driven by perceived susceptibility, perceived severity, perceived benefits, perceived barriers, cues to action, and self-efficacy [24]. When symptoms are minimal and artificial tears fail to provide immediate relief, perceived susceptibility and perceived benefit may be reduced, resulting in diminished motivation. Both models suggest that improving compliance requires external prompts targeting capability and opportunity, and a rationale to act that reshapes motivation and health beliefs.

Mobile health (mHealth) applications (apps) can deliver such prompts, improve treatment compliance when combined with self-monitoring and feedback, although effects are often modest and not sustained without ongoing engagement features [25,26,27]. In ophthalmology, app-based reminders have improved adherence in glaucoma and allergic conjunctivitis [28,29]. For example, Leshno and co-authors [30] showed that persistent auditory alerts markedly improved glaucoma adherence, shifting patients from low to high compliance. Ghosh and colleagues [31] identified customisable reminders, goal setting, rewards, social connection, and clinician data sharing as features that sustain engagement. Habit formation, which underpins long-term adherence, requires repeated behaviour in a stable context over time [32,33]. In DED, the DryEyeRhythm platform has recorded widespread drop underuse [34], but whether reminders can change compliance behaviour remains untested.

To date, no randomised trial has examined whether smartphone reminders improve artificial tear compliance in adults with DED. Using a prospective crossover design, this study, informed by patient feedback, tested whether the reminder app increased daily drop instillation compared with a control period without reminders. Secondary objectives were to assess symptom frequency and severity and to examine whether baseline clinical measures predicted compliance. It was hypothesised that reminders would increase drop use, but that the effect would be limited by the short intervention period and the app’s current limited engagement features.

## 2. Materials and Methods

This was a prospective, single-masked, randomised crossover trial (registration clinical trials.com NCT06163989, 1 September 2024) comprising two 14-day phases, one with app reminders and one without, separated by a 7-day washout period (of standard dry eye care) [35]. It was conducted from a university eye clinic in Birmingham, United Kingdom, from October 2025 to March 2026. The participant flow and crossover sequence are shown in Supplementary Figure S1.

### 2.1. Participants

Participants were eligible if they were aged 18 years or older. Participants who expressed interest (*n* = 36) were sent the Ocular Surface Disease Index (OSDI) 6 questionnaire [36] electronically to complete. Those who obtained an OSDI score of 4 or more were invited to attend a baseline visit to confirm the presence of a non-invasive breakup time < 10 s, >5 corneal fluorescein, or >9 conjunctival lissamine green punctate spots to confirm the TFOS DEWS III diagnosis of DED criteria [6].

Corneal staining was evaluated following application of a saline moistened strip of Sodium Fluorescein, illuminated with a blue light and observed through a yellow filter under 10× magnification (Antares + topographer (CSO, Florence, Italy) [37]. Participants were instructed to look in the four gaze positions (superior, inferior, nasal, and temporal) and any superficial punctate staining observed was counted. Conjunctival staining was then assessed with lissamine green dye with a drop of saline placed on the strip, with the whole drop applied to the temporal canthal area whilst the patient was in an upwards gaze, after 10s. The patient was then told to blink after instillation to distribute the dye evenly across the ocular surface. The conjunctiva was viewed one to four minutes after instillation [38].

Exclusion criteria were inability to run the reminder app on their personal smartphone, ocular surface disease other than dry eye, unstable systemic disease likely to impact the ocular surface, and any dry eye treatment other than artificial tears in the previous 6 months. Twenty nine applicants met the eligibility criteria and were included (mean ± SD age 21.2 ± 3.0 years; all female).

The study adhered to the Declaration of Helsinki and was approved by the Aston University Health and Life Sciences Ethics Committee (Optometry Panel). All participants provided written informed consent.

### 2.2. Procedures

The reminder app was set up on each participant’s smartphone with reminder times chosen by the individual. The participant was given an envelope with instructions on whether to activate the app for the first or second 14-day phase generated by an investigator not involved in the clinical assessments using a random number generator.

Participants were assessed, by a masked investigator, consistently in the morning or early afternoon to limit the influence of diurnal variation, as DED symptoms tend to worsen in the evening following prolonged visual display unit use [39]. Participants were also assessed between November and February, during the UK winter, to reduce potential seasonal effects. All assessments were conducted in the same air-conditioned examination room, with the testing environment kept the same by controlling temperature and illumination.

The primary outcome was daily drop frequency, recorded from the app digital log (during the app use phase) or via a paper diary (during the non-app phase) on a daily basis. Secondary outcomes were daily Symptom Assessment iN Dry Eye (SANDE) [40] frequency and severity scores throughout the intervention phases. Baseline OSDI-6, NIBUT, and staining scores were examined as potential predictors of mean drop use and app-related improvement. A history and symptom review was conducted at each visit to identify adverse events.

### 2.3. Statistical Analysis

A power calculation (G*Power v3.1.9.7) identified that 95% power with an effect size of 0.25 and a significance level of *p* < 0.05 could be achieved with 18 participants. No participants had missing data and all completed both the app and no-app study phases.

As the data was normally distributed, repeated-measures ANOVA was used to assess the effects of two within-subject factors, phase (app, non-app) and day (1–14). Multivariable linear regression examined whether baseline measures predicted mean drop use or app-related improvement. Analyses were performed using SPSS version 29.0 (IBM, Armonk, NY, USA).

## 3. Results

The baseline findings were consistent with mild-to-moderate DED: the mean OSDI-6 score was 8.1 ± 5.1 (range 4–23), mean non-invasive breakup time was 8.0 ± 3.6 s (3.0–18.2), the mean number of corneal punctate spots was 24.9 ± 36.0 (0–175), and the mean number of conjunctival punctate spots was 53.6 ± 75.6 (0–305). No adverse events were reported.

**Figure 1:**
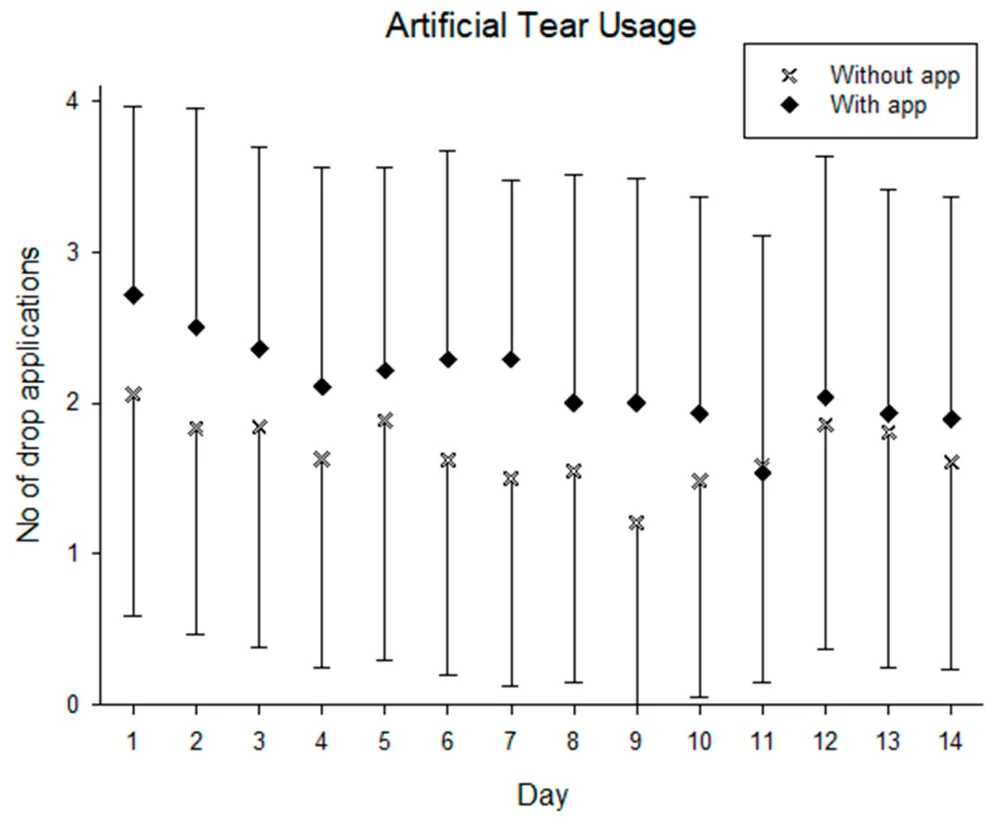
Mean daily drop frequency during the app and non-app phases over the 14-day study period. Error bars = 1 standard deviation.

Drop use frequency was higher during the app phase than during the non-app phase (*F* = 22.906, *p* < 0.001), but declined over time in both phases of the trial (*F* = 3.023, *p* < 0.001; Figure 1) with a similar pattern (phase-by-day interaction: *F* = 1.235, *p* = 0.252).

**Figure 2:**
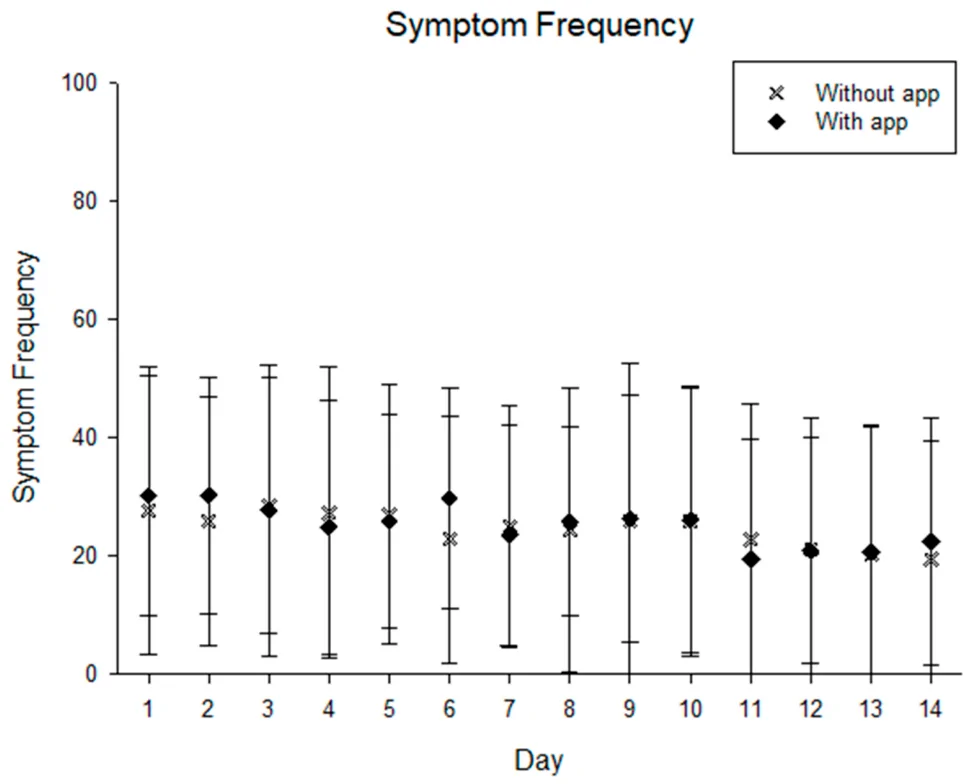
Mean daily symptom frequency scores during the app and non-app phases over the 14-day study period. Error bars = 1 standard deviation.

**Figure 3:**
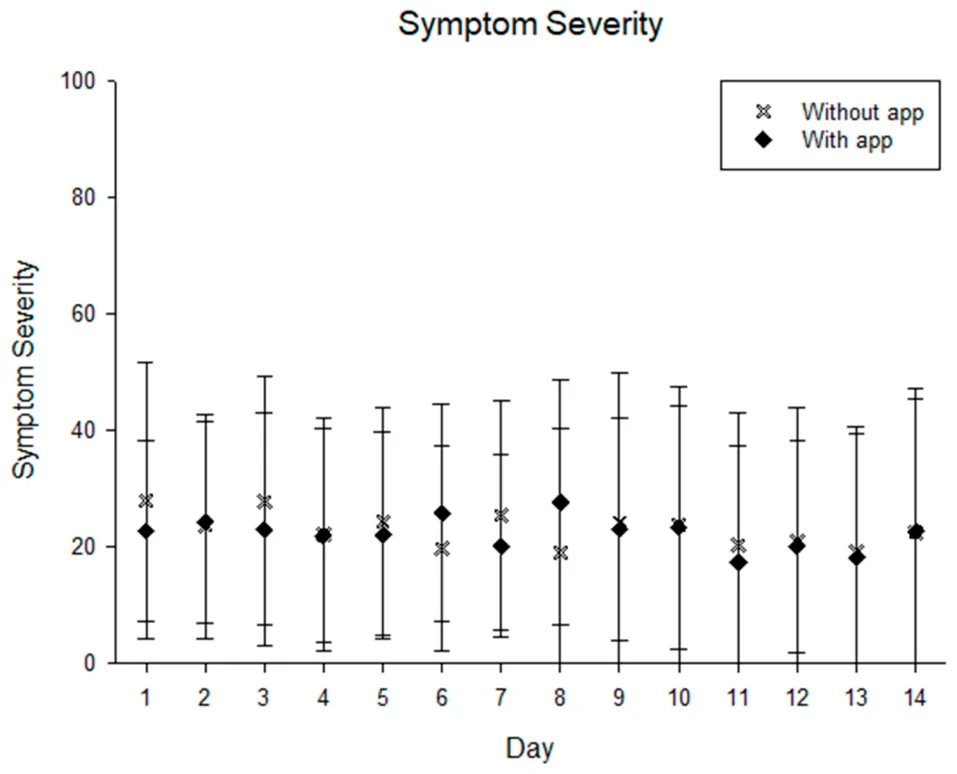
Mean daily symptom severity scores during the app and non-app phases over the 14-day study period. Error bars = 1 standard deviation.

Symptom frequency did not differ between phases (*F* = 0.116, *p* = 0.736), but decreased over time with the app and no-app phases (*F* = 1.993, *p* = 0.021; Figure 2). Symptom severity was not affected by phase (*F* = 0.146, *p* = 0.705) or time (*F* = 0.920, *p* = 0.532; Figure 3).

In a multivariate analysis, the baseline clinical measures did not predict drop frequency (*F* = 0.642, *p* = 0.671) or app-related improvement (*F* = 0.547, *p* = 0.738). Both models explained less than 10% of the variance. There was no difference in the improvement in drop usage with the app between those who were already using artificial tears (*n* = 13) prior to the study than those who only used them in the study phases (+0.44 ± 0.35 vs. +0.47 ± 0.61, *p* = 0.888).

## 4. Discussion

The reminder app increased short-term compliance by, on average, 0.5 doses per day. While this was not enough to significantly improve symptoms over the two weeks of use, prior research suggests longer-term usage of artificial tears is needed to improve ocular signs [14], so any improvement in compliance may be beneficial. Interestingly, drop use declined over the fortnight following the initial appointments in each phase, regardless of active reminders being used or not.

The findings fit the COM-B and HBM behavioural frameworks. The app successfully targeted opportunity by delivering a salient time-contingent prompt and supported psychological capability by removing the need to remember [23]. It did not alter reflective motivation or perceived benefit [24]. Without an increase in perceived benefit and a sustained sense of susceptibility, behaviour returns to baseline and is unlikely to be maintained [24]. The progressive decline in drop use while reminders were still active confirms prompts alone are insufficient, particularly when educational content and features supporting long-term engagement and habit formation are absent [32,33].

The absence of symptom relief was expected; participants did not reach the four-times-daily threshold recommended for meaningful symptom improvement, which requires at least one month of regular use [14,19]. An extra half-dose per day is marginal and the two-week intervention was too brief for therapeutic tear film changes to occur. All participants used the same lipid-supplemented artificial tears regardless of DED drivers [6]. Individuals with lipid-, aqueous-, and mucin-deficient tear films may respond differently to specific formulations [14,41], so some participants may have experienced minimal physiological benefit despite using the drops, reducing the reinforcement needed to strengthen the dosing habit.

The size of the effect is consistent with the broader mHealth literature. Thakkar and colleagues [42] pooled text-message reminder trials across cardiovascular, respiratory, and metabolic diseases, and reported an adherence gain of 11.3%. When applied to a four-times-daily regimen, this translates to roughly 0.5 extra doses per day, matching what was observed between app and non-app phases. A simple prompt, whether an SMS in cardiovascular disease or push notifications in dry eye, shifts behaviour by a similar amount. Larger effects in ophthalmology have required more intensive interventions. Leshno and colleagues [30] used persistent auditory alerts in glaucoma that lasted until the patient confirmed instillation, yielding a greater adherence gain. The reminder app deployed standard push notifications which could be easily ignored or missed, highlighting that the intensity of the prompt matters.

Long-term compliance depends on how well an intervention supports engagement in everyday life [25]. The app supplied reminders and weekly compliance summaries, elements that can initiate use but generate little intrinsic motivation. The absence of goal setting, rewards, social connection, and clinician-mediated feedback, identified as critical for long-term mHealth engagement [31] likely contributed to the observed decay in adherence. A notification alone does not provide the stable context needed for repeated behaviour to become automatic [32,33].

Clinically, a reminder app can help patients whose sole barrier is forgetfulness, but has minimum effects for patients who do not understand why drops are needed when their eyes are already reasonably comfortable. Patients therefore require education in which it is explained that artificial tears are preventive, rather than just reactive [21,22,43].

By utilising a crossover design, the study effectively controlled for individual confounding variables including age, baseline motivation, symptom severity, and digital literacy that typically weaken parallel group comparisons. The investigators were masked to the phase order, reducing potential expectation bias. All tests were completed in the same room, at the same time of day, controlling for diurnal and seasonal tear film variability. DED was diagnosed using TFOS DEWS III criteria [6], ensuring participants had both symptoms and objective signs of DED rather than a symptomatic overlay alone. A younger, digitally literate sample was intentionally selected. If push reminders are insufficient to enhance compliance in this group, it is unlikely that the app would achieve greater adherence in older adults, for whom technological unfamiliarity may represent an additional barrier.

A limitation is that medication compliance was not evaluated directly. During the intervention phase, an assumed dose was recorded automatically whenever a participant tapped the push notification; however, a digital acknowledgment does not equate to successful clinical instillation. Conversely, during the non-application phase, participants relied exclusively on paper diaries to log their dosing. This methodological disparity between digital tracking and paper-based logs may have introduced measurement and reporting bias. Self-reported adherence is susceptible to recall and social desirability bias, with estimates often exceeding objectively monitored adherence by 20–30% [44]. Consequently, the true discrepancy in compliance between phases may be wider than reported.

Additionally, the 7-day washout period may have been insufficient to fully eliminate carry-over effects or disrupt behavioural routines established in the preceding crossover arm.

Furthermore, the two-week intervention window may have been too brief to facilitate authentic habit formation or yield meaningful physiological alterations in the tear film.

The participants were young women recruited from a university setting. These findings may not be applicable to male populations, older cohorts, or individuals with severe DED. As all participants used the same lipid-based artificial tear drop, it is not possible to differentiate between the app’s behavioural effects and how well that precise formula worked for different types of dry eye. Finally, by not evaluating patients’ initial views about their disease or treatment expectations, known to impact long-term habits [45], the underlying psychological reasons why their compliance diminished over time are unknown.

## 5. Future Directions

Future research should employ objective electronic drop dispense monitoring in both arms over a longer period to determine whether the 0.5 dose gain persists or decays further. An enhanced application incorporating persistent alerts, context-aware triggers, goal settings, and/or embedded education targeting the motivational barriers identified by the COM-B and HBM model should be developed and tested. Qualitative research with DED patients of all ages and severity levels would help to define the personal disease models that motivate or restrict adherence. Digital tools will be most effective when integrated into a comprehensive care pathway that includes correct drop formulation matched to disease subtype, explicit preventive education, and regular clinical evaluation.

## 6. Conclusions

The reminder app yielded a modest, statistically significant, short-term increase in artificial tear compliance among young adults with dry eye disease. However, this behavioural improvement did not provide additional symptoms relief and declined over time despite active reminders. Prompts alone are insufficient to sustain adherence. Future digital interventions for DED should integrate features that build motivation and support long-term engagement, delivered alongside individualised patient education.

## Data Availability

The raw data supporting the conclusions of this article will be made available by the authors on request.

## Supplementary Materials

The following supporting information can be downloaded at https://www.mdpi.com/article/10.3390/jcm15166369/s1, Figure S1: Study schema illustrating participant screening, randomisation to crossover sequence, the two 14-day study phases, 7-day washout period, and completion through final analysis. Shaded boxes denote the app phase. All 29 randomised participants completed both phases; no attrition occurred after randomisation.
