# Supplementary material for: The Impact of a Smartphone Reminder Application on Artificial Tear Adherence in Dry Eye Disease
Source: J Clin Med. 2026 Aug 18;15(16):6369. doi: 10.3390/jcm15166369 (PMC13513040; doi:10.3390/jcm15166369)
Supplement: Supplementary file 1 [file jcm-15-06369-s001.zip › jcm-4451583-supplementary.pdf]

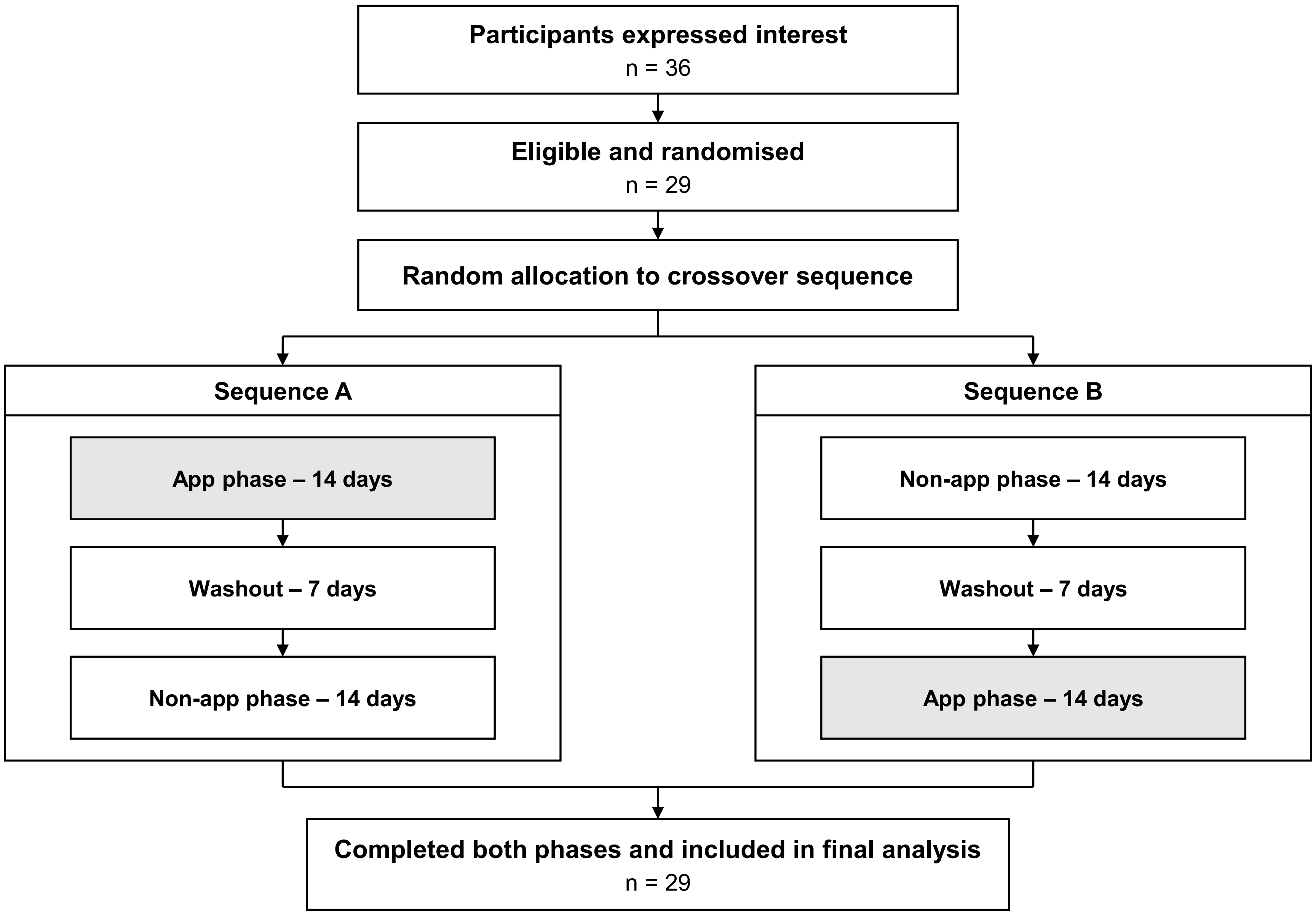

Figure S1. Study schema illustrating participant screening, randomisation to crossover sequence, the two 14-day study phases, 7-day washout period, and completion through final analysis. Shaded boxes denote the app phase. All 29 randomised participants completed both phases; no attrition occurred after randomisation.
